# Glucose Gel as a Potential Alternative Treatment to Infant Formula for Neonatal Hypoglycaemia in Australia

**DOI:** 10.3390/ijerph15050876

**Published:** 2018-04-27

**Authors:** Raenee L. Barber, Amy E. Ekin, Pushparani Sivakumar, Kay Howard, Therese A. O’Sullivan

**Affiliations:** 1 School of Medical and Health Science, Edith Cowan University, Joondalup 6027, Australia; raenee.barber@gmail.com (R.L.B.); amyekin@gmail.com (A.E.E.); 2 Nutrition and Dietetics Department, King Edward Memorial Hospital, Subiaco 6008, Australia; Pushparani.Sivakumar@health.wa.gov.au; 3 Murdoch University, School of Veterinary and Life Sciences, Murdoch 6150, Australia; kay.howard@iinet.net.au

**Keywords:** Glucose gel, hypoglycaemia, infant formula, neonate, diabetes, breastfeeding

## Abstract

Infant formula is often used as a treatment for neonatal hypoglycaemia in Australia; however, there are concerns that this may jeopardise mother-baby bonding and breastfeeding. Successful use of glucose gel as an alternative treatment for hypoglycaemia has been reported. We wanted to investigate in a pilot study whether the use of glucose gel has the potential to quickly and safely restore normoglycaemia in the infants of diabetic mothers in an Australian setting. Infants with asymptomatic hypoglycaemia were treated with glucose gel (*n* = 36) and compared to a historical group of infants which had been treated with infant formula (*n* = 24). Within 15 min of the first treatment, the gel group had a mean blood glucose level (BGL) of 2.6 mmol/L, and 2.7 mmol/L 30 min after the second treatment. This was lower than the BGL after the first treatment for the formula group, which rose to a mean of 2.8 then to 3.2 mmol/L after the second treatment (*p* = 0.003). In successfully treated infants, administration of the gel resulted in normoglycaemia within 30 min. The likelihood of special care nursery admission was not significantly different between the groups, although we had a small sample size, and our findings should be interpreted with caution. These pilot results provide support for further investigations into the use of glucose gel as an alternative treatment to infant formula.

## 1. Introduction

Transient, asymptomatic low blood glucose concentrations occurs frequently in newborns, and may play a role in the stimulation of postnatal glycogenolysis and gluconeogenesis [1], development of appetite, and the adaption to fast/feed cycles [2]. However, asymptomatic hypoglycaemia may also indicate underlying metabolic disorders that can cause serious injury [2]. Hypoglycaemia in at-risk groups, such as babies of diabetic mothers, can result in motor and neurological dysfunction if not properly treated [3,4,5]. Therefore, it is imperative that nurseries are vigilant, and provide prompt, safe treatment for all infants until it is determined that they can maintain normal glucose concentrations through fast-feed cycles [2].

Unfortunately, there is currently a lack of consensus regarding the best method of treatment for neonatal hypoglycaemia [3,6,7,8]. Within Australia and New Zealand (NZ), there is wide variation in the treatment of asymptomatic hypoglycaemia within and between nurseries. Recent reviews have indicated that there is a paucity of studies to provide the type of data needed to establish a definitive approach [9,10]. Furthermore, it is difficult to compare results of previous studies due to the different types of blood glucose level (BGL) measurements taken (interstitial/continuous/heel-prick blood samples), the accuracy of the measuring device used, the timing of treatments [11], the subsequent timing of the BGL measurement [11], and the influence of the risk factors of the participants.

As maternal breast milk supply can often take a few days to become established, there is a need for a treatment other than breast milk to be available. In Australia and NZ, infants who develop asymptomatic hypoglycaemia, indicated by a BGL between 2.0 and 2.5 mmol/L, are most commonly treated with infant formula [8,12]. However, midwives are concerned that treatment with infant formula may jeopardise mother-baby bonding and the successful establishment of breastfeeding [13]. Alternative treatments therefore need to be investigated.

A potential alternative treatment is the sublingual administration of a 40% glucose gel [8,14]. At about US$2 per patient [15], or AU$4 in Australia, there is evidence that glucose gel is more effective in raising BGL than breastfeeding alone [16,17,18,19,20,21]. The sublingual area has a thin, permeable epithelial structure, with blood vessels draining directly into the jugular vein and moving quickly into the systemic circulation, bypassing the hepatic first-pass metabolic processes. This provides improved bioavailability over oral dosing [22]. The advantage of using the sublingual surface over the buccal area is that it limits the amount of glucose to be absorbed, due to the saturability of the glucose carriers [23]. A rapid but steady increase in BGL should reduce the incidence of rebound hypoglycaemia.

There are increasing numbers of diabetic mothers, as gestational and Type 2 diabetes rates continue to rise throughout the world [24]. Hypoglycaemia is common in infants born to diabetic mothers, as they are generally exposed to elevated maternal blood glucose levels during pregnancy. This can lead to increased neonatal insulin levels and rapid use of available energy stores after birth, placing them at high risk of hypoglycaemia. Almost half of Australian and NZ nurseries reported that they did not treat the babies of diabetic mothers in the first three hours after birth, other than to breastfeed [8]. Given there are also doubts on formula being used as a treatment for at-risk infants with asymptomatic neonatal hypoglycaemia in Australia, carefully designed clinical investigations are required to provide reliable evidence for the best clinical practice for the treatment of hypoglycaemia [8]. In an effort to contribute towards an important piece of this multifaceted puzzle, we investigated the use of glucose gel, in conjunction with breastfeeding, for asymptomatic hypoglycaemia in infants of diabetic mothers in a West Australian maternity hospital.

We undertook a pilot study of glucose gel treatment with matched historical records in order to determine the feasibility of this treatment for future, larger studies. Our aim was to compare infants who were treated with glucose gel with retrospective data of infants who were treated with infant formula, to determine if glucose gel was as effective in rapid establishment of normoglycaemia and prevention of special care nursery admissions.

## 2. Materials and Methods 

This pilot study was conducted between August 2011 and June 2012 at King Edward Memorial Hospital, Subiaco, Australia, to test the ability of glucose gel to normalise BGL (≥2.6 mmol/L).

### 2.1. Subjects

Infants born to diabetic mothers from ≥36 weeks gestation with asymptomatic hypoglycaemia were eligible for inclusion. Asymptomatic hypoglycaemia is seen in otherwise healthy infants with a BGL of between 2.0–2.5 mmol/L prior to the second breastfeed [6]. Recruitment of infants took place at the antenatal clinic, whereby pregnant women (>18 years old) with diabetes (gestational, Type 1 or Type 2) were approached. An information letter explaining the study was provided, and a signed consent form collected. Inclusion of infants was only possible if they were admitted to a ward with an onsite blood gas analyser, resulting in a convenience sample of infants admitted to the postnatal ward. Infants were excluded if they were symptomatic, had a BGL below 2.0 mmol/L, had any other health issues, were not being exclusively breastfed, or if they were offered formula at any point during the study. 

Retrospective data from medical records (from 1 March 2011 to 31 July 2011) was used for the formula group. The historical controls were breast fed infants, with formula subsequently used in the treatment of hypoglycemia. The same inclusion/exclusion criteria were applied, except that these infants were treated with formula. The same measurements were taken for BGL, using the VITROS^®^ system (Ortho Clinical Diagnostics, NJ, USA).

A good match of characteristics of participants was achieved between the gel and the formula groups (Table 1), with no significant difference (*p* > 0.05) in infant gender, weight, gestational age or maternal age. However, there was a significant difference (*p* = 0.03) in the type of maternal diabetes, with the gel group having 25% less mothers with gestational, and 2.8 and 5.5 times more Type 1 and Type 2 diabetes respectively, compared to the formula group. 

### 2.2. Protocol

**Figure 1:**
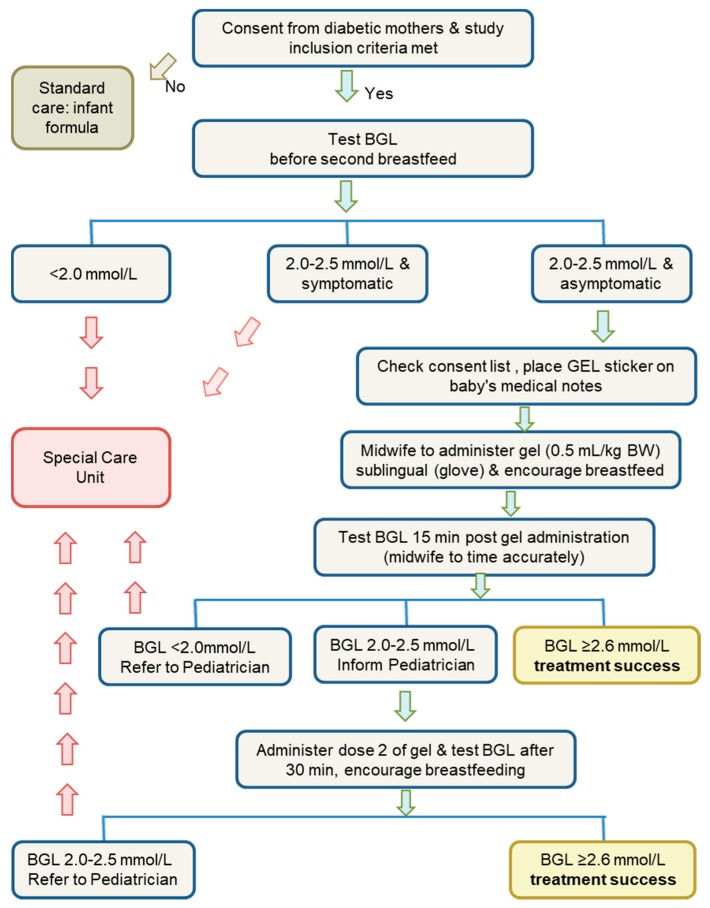
Protocol for selection and treatment of infants, of diabetic mothers, suffering hypoglycaemia for trial of buccal application of glucose gel treatment. BGL: blood glucose levels, BW: body weight

A flow chart of the methodology used is shown in Figure 1. Glucose concentrations were determined using the glucose oxidase method with blood gas analyser (ABL 90 Flex, Radiometer, California, USA) via whole blood capillary heel prick lancing [6]. For the gel group, BGL was determined at 15 min post treatment 1, and at 30 min if a second dose of the glucose gel was administered. Treatment was considered successful if the infant’s BGL reached ≥2.6 mmol/L. The formula group followed the Figure 1 protocol, with the BGL processed in the on-site hospital laboratory. Due to the use of historical data as a control group, it was not possible to tightly control protocol, and there were likely to be differences in the timing of feeds and testing.

All infants treated with glucose gel were exclusively breastfed, and BGL was tested prior to the second breastfeed (within four hours of birth), in order to diagnose hypoglycaemia. Continued breastfeeding was encouraged throughout the treatment period. Initial treatment for the hypoglycaemic infant was a 40% dextrose gel (Glutose 15, Paddock Laboratories, Minneapolis, MN, USA). A 2 mL sterile syringe measured a dosage of 0.5 mL/kg of body weight [20]. The glucose gel was administered sublingually with a gloved hand, after sterile gauze was used to first dry the area to enhance absorption. The midwives were responsible for administering the glucose gel. They participated in two one-hour training sessions where the correct technique in applying the glucose gel was demonstrated. However, it was realized within the first few weeks that there was often not enough space to apply the dose sublingually, so any remaining gel was massaged into the buccal membrane.

### 2.3. Approval

The study was approved by The Women’s and Newborn Health Service Ethics Committee (1905/EW) and The Edith Cowan University Human Research Ethics Committee (6541).

### 2.4. Statistical Analysis

Statistical analysis was performed using Predictive Analytics Software for Windows, version 18.0 2009 (SPSS Inc., IBM, Chicago, IL, USA). The means of both treatment groups were compared using an independent t test. A Chi Squared test of contingencies, with a significance level of *p* < 0.05, was used to compare treatments with regard to special care nursery admission.

## 3. Results

### 3.1. BGL Response to Treatments

**Figure 2:**
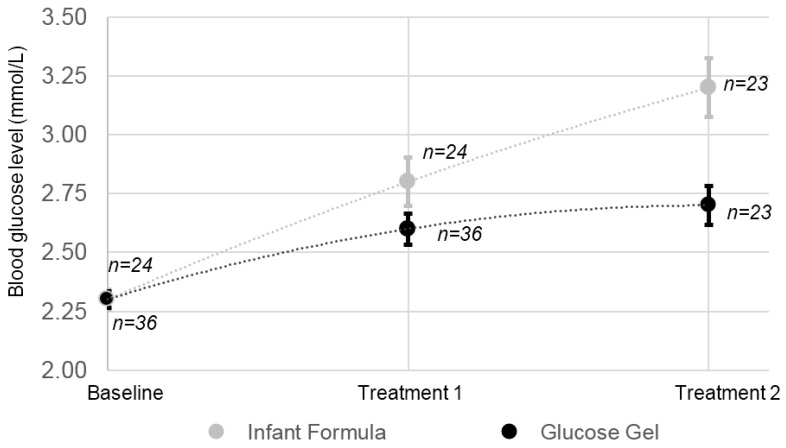
Means of blood glucose response to either infant formula or glucose gel treatment in hypoglycaemic infants born to diabetic mothers. Error bars represent standard error. Blood glucose levels (BGL) were measured 15 min after the first treatment; where required, a second treatment was administered, then the BGL was measured again after 30 min.

Starting with the same mean baseline BGL of 2.3 ± 0.2 mmol/L, both treatment groups saw a rise of BGL following the first treatment (Figure 2). While the mean of the gel group reached the desired mark of 2.6 mmol/L for normalisation of BGL, the mean of the BGL in the infant formula group rose above this (*p* = 0.07). The mean BGL of the infant formula group continued to increase after the second treatment, reaching a mean of 3.2 ± 0.6 mmol/L, and creating a significant difference between the groups (*p* = 0.003). The gel group had a smaller variance in the BGL after both treatments, compared to the formula group. In the gel group, 64% of infants were administered a second treatment, while 96% of the infant formula group were administered a second treatment (Figure 2). 

### 3.2. Time to Normoglycemia

In successfully treated infants in the glucose gel group, all had reached normoglycemia within 30 min.

### 3.3. Special Care Nursery Admission

Ninety-six percent of the formula group—compared to 81% of infants treated with glucose gel—avoided special care admission. This difference was not determined to be significant (𝜒^2^ = 0.2; *p* = 0.08). A retrospective power calculation (percentage, two sample, one-tailed) found that our study had 52.2% power to detect whether the treatment group had a significantly higher rate of admission to special care than the control group, at the 0.05 level [25].

### 3.4. Adverse Reactions

No infants had an adverse reaction to the glucose gel.

## 4. Discussion

The results of this pilot study contribute to the establishment of a new standard of care for neonatal hypoglycaemia. Our findings suggest that the use of glucose gel is worth investigating as an effective treatment for hypoglycaemia in Australia. If successful, this treatment has the potential to contribute to a reduction in related lengths of hospital stays and costs [10,20,26,27]. Although our results showed that special care nursery admission, in infants diagnosed with asymptomatic hypoglycaemia, did not significantly differ with treatment of glucose gel or infant formula, based on the differences detected and our final sample sizes, results may be due to being under-powered rather than genuinely demonstrating no difference between groups. Special care nursery admission is generally detrimental to bonding and establishment of breastfeeding, and is highly stressful for parents and neonates [10]. Mothers in continuous contact with their infants are found to produce more milk, breastfeed longer, and are more likely to exclusively breastfeed [28].

Glucose gel treatment resulted in a quick establishment of normoglycaemia (within 15 min) for 36% of infants. This supports findings of other researchers: Harris et al. measured at 20 min [29]; Harris et al. at 10 min (sugar powder) [20], while Ter et al. found an increase at 10 min, with a peak at 40 min [21]. Our results showed that a higher percentage of infants in the formula group received a second treatment. The measurement of BGL for the formula group in our study was conducted in the hospital laboratory rather than being ward based, and there may have been some delays in receiving results which lead to unnecessary second treatments. Although our results support formula as an effective treatment for hypoglycemia, there remains little evidence on the time to peak BGL for formula treatments, and no evidence-based guideline for the volume of formula required for treatment [16]. Our first gel treatment resulted in a mean BGL of 2.6 mmol/L, with low variance in the group. This more gradual change in BGL produced by glucose gel was also reported by others [30], and a normal range of BGL achieved, without rebound or recurrent hypoglycaemia [20,23,27,29] and no hyperglycaemic reactions [27]. Importantly, there were no adverse effects from using gel, as reported in previous studies [9,20,31].

Our study showed that infant formula is also an effective treatment, resulting in the greatest increase in BGL, also reported by Harris et al. [16]. The second treatment in the current study resulted in an average BGL of 3.2 mmol/L, significantly higher than the gel results. However, a higher BGL is not necessarily better, as higher post-treatment plasma glucose in infants with neonatal hypoglycaemia has been associated with poor neurodevelopment at two years of age, suggesting a ‘U’-shaped relationship between neonatal glucose concentrations and neurodevelopmental impairment [32]. Higher BGLs are more likely to result in rebound hypoglycaemia [23].

The importance of breastfeeding infants—due to its immunological and nutritional value—is scientifically supported by multiple organisations, including the World Health Organisation [33]. However, with many hospitals still promoting formula as a treatment for hypoglycaemia, there are many concerns that this may contribute to lower breastfeeding duration [13,34]. Disruption of the establishment and duration of breastfeeding can result in increased risk of infections and allergies, and alterations in the neonatal microbiome [16]. The use of infant formula may decrease a mother’s confidence in her own breast milk and breastfeeding ability, potentially reducing the chance of successful breastfeeding, and also the duration of breastfeeding [34,35]. This is of particular importance in our growing population of diabetic mothers, as mothers who continue breastfeeding decrease the risk of their child experiencing metabolic disorders later in life [35,36]. A consistent result from studies on the use of glucose gel is the improvement in exclusive breastfeeding rates [10,20,27,30,37].

The use of the glucose gel dose is less invasive than IV treatment and less disruptive to breastfeeding than formula; however, sublingual application proved difficult in some infants where the area for application was very small. As a result, some applications were sublingual with the remainder massaged into the buccal membrane. This may result in some of the gel being swallowed. Swallowing of the gel decreases the bioavailability of the glucose, due to reliance on gastrointestinal and hepatic metabolism, as opposed to the direct passage to the systemic circulation if absorbed [38]. This could be overcome by more frequent sublingual applications of gel. 

Administration of a small amount of sucrose for pain relief prior to BGL testing [39] was an exclusion criteria for this study, however this may not have been necessary. A Cochrane review on this topic [40] found that the only study to investigate effect of sucrose on BGL found no significant difference in BGL between babies to whom sucrose had been administered, compared to those given a placebo (sterile water) in a double blind, randomized controlled trial [41]. Future studies may decide to omit this as an exclusion criterion.

The type of maternal diabetes differed between gel and formula groups in our study, with the formula group having a higher proportion of gestational diabetes and lower proportions of Type 1 and Type 2 diabetes. In all types of diabetes, blood glucose control is key to minimizing adverse effects; therefore, it is difficult to say whether the type of diabetes would make a difference to our findings. It is also possible that some mothers with gestational diabetes may have already had undiagnosed Type 2 diabetes. 

The use of glucose gel with breastfeeding was associated with reduced requirement for repeat gel treatment. Research indicates that breastfeeding may have a slower, more sustained effect on BGL than infant formula or dextrose gel [16]. Glucose gel supplies a rapidly absorbed and digested carbohydrate, and is therefore useful as a first line treatment, followed up by breast milk as a slower-acting carbohydrate [16]. The use of glucose gel has been shown to increase breastfeeding rates upon discharge from hospital, compared to other treatments; nonetheless, larger studies are required to empirically test the long-term effects on breastfeeding. Our study provides support for a large-scale, Australian randomized controlled trial to provide more definitive data on the treatment of hypoglycemic infants. In terms of preventing hypoglycaemia for infants at risk (including preterm, small or large, as well as having a mother with diabetes), a randomized, multicenter, placebo controlled trial is underway in Australia and NZ [42]. This trial plans to investigate whether a preventative dose of gel can reduce admission to special care along with improving breastfeeding and maternal satisfaction. 

## 5. Conclusions

Our results support previous research in other countries, indicating that sublingual/buccal glucose gel is a promising treatment in infants diagnosed with asymptomatic hypoglycaemia. The glucose gel treatment provided rapid and controlled improvements in BGL. The formula group had a larger increase in BGL with greater variance in response. There were no adverse reactions to glucose gel, and we did not detect significant differences in special care nursery admissions between the two groups, although we were underpowered for this outcome. Our results must be interpreted with caution, given our low subject numbers. While some individual Australian hospitals have developed protocols utilizing glucose gel treatment, many do not. Further research into glucose gel treatment is required to justify development of uniform policies across the region.

## Figures and Tables

**Table 1:** Characteristics of hypoglycaemic infants born to diabetic mothers from both the glucose gel treatment group and the historical infant formula group.

	Mean ± Standard Deviation		
	Infant Formula (*n* = 24)	Glucose Gel (*n* = 36)	*p* Value
**Gender (male)**	12 (50%)	20 (55%)	0.68
**Weight (g)**	3330 ± 392	3496 ± 518	0.16
Range	2870–4440	2665–4160	
**Gestational age (weeks)**	38 ± 0.72	38 ± 1.12	0.27
Range	37–40	36–40	
**Maternal age (years)**	33 ± 6	38 ± 1	0.27
Range	22–45	21–45	
**Type of maternal diabetes**			
Gestational	22 (92%)	24 (67%)	0.03
Type 1	1 (4%)	4 (11%)	
Type 2	1 (4%)	8 (22%)	
